# Comparison of the effects of deferasirox film-coated tablets (Jadenu^®^) and deferasirox dispersible tablets (Exjade^®^) in patients with beta thalassemia major: a preliminary report of the effects on the satisfaction, convenience, cardiac/liver MRI T2*, serum ferritin level, and biochemical profiles

**DOI:** 10.3389/fphar.2024.1438611

**Published:** 2024-11-19

**Authors:** Mahya Mobinikhaledi, Vahid Falahati, Amin Tajerian, Amir Almasi Hashiani, Kazem Ghaffari, Ali Ghasemi

**Affiliations:** ^1^ Faculty of Medicine, Arak University of Medical Sciences, Arak, Iran; ^2^ Department of Pediatrics, Faculty of Medicine, Arak University of Medical Sciences, Arak, Iran; ^3^ Department of Epidemiology, School of Health, Arak University of Medical Sciences, Arak, Iran; ^4^ Hematology and Transfusion Science Department, School of Allied Medical Sciences, Tehran University of Medical Sciences, Tehran, Iran; ^5^ Department of Basic and Laboratory Sciences, Khomein University of Medical Sciences, Khomein, Iran; ^6^ Department of Biochemistry and Hematology, Faculty of Medicine, Semnan University of Medical Sciences, Semnan, Iran

**Keywords:** thalassemia major, deferasirox, liver, heart, iron overload, satisfaction, convenience

## Abstract

**Background:**

Deferasirox (DFX) is a once-daily oral iron chelator with proven dose-dependent efficacy in patients with thalassemia major (TM). The reason for switching from DFX dispersible tablets (Exjade^®^) to DFX film-coated tablets (Jadenu^®^) was intolerance. Many patients also reported that deferasirox^®^ did not taste good. In this study, we compared the effect of Jadenu^®^ and Exjade^®^ on satisfaction, convenience, cardiac/liver MRI T2*, serum ferritin levels, and biochemical profiles in patients with thalassemia major.

**Method:**

Sixty-two patients with thalassemia over 2 years of age, who had iron overload indicated by chelation therapy, were randomly divided into two groups. The first group (n = 32) is treated with Exjade^®^, and the second group (n = 30) is treated with Jadenu^®^. Laboratory investigations included alkaline phosphatase (ALK), alanine transferase (ALT), aspartate transferase (AST), and serum ferritin levels. Cardiac/liver MRI T2* levels and patient satisfaction and convenience, were assessed before and 1 year after starting therapy.

**Results:**

The study found that 53.3% of Jadenu^®^ patients were satisfied with the taste of the medication compared to only 12.5% of Exjade^®^ patients, which was statistically significant (*p* = 0.001). Additionally, 40% of Jadenu^®^ patients were satisfied with the ease of taking the medication compared to 28.1% of Exjade^®^ patients, and again, the difference was statistically significant (*p* = 0.047). A comparison of the cardiac MRI T2* levels between the two studied groups showed no significant difference (*p* = 0.851).

**Conclusion:**

Jadenu^®^ offers patients an improved formulation that can be taken on an empty stomach, has a better taste, and presents fewer gastrointestinal tolerability concerns. Overall, patient satisfaction is higher with Jadenu^®^, which may improve adherence and reduce the frequency and severity of complications associated with iron overload. This, in turn, may help mitigate cardiovascular and hepatic complications from iron overload in the long term.

**Clinical Trial Registration:**

https://irct.behdasht.gov.ir/search/result?query=IRCT20210830052346N1

## Introduction

Mutations in the genes encoding alpha- or beta-globin chain synthesis cause thalassemia, a common genetic disorder with autosomal recessive inheritance that ultimately leads to severe microcytic hypochromic anemia and ineffective erythropoiesis (Muncie and Campbell, 2009; Eghbali et al., 2023a). The high prevalence of this disorder can be found in the Mediterranean, Africa, Southeast Asia, and the Middle East (Old et al., 2005; Ghasemi et al., 2023). Individuals diagnosed with thalassemia major (TM) require regular blood transfusions for survival, with each milliliter of packed red blood cells contributing to a milligram increase in iron levels in TM patients undergoing transfusions (Khalifa et al., 1985). As a result, iron overload is a significant long-term consequence of frequent blood transfusions that puts recipients at risk for heart disease, liver disease, and endocrine abnormalities (Borgna-Pignatti and Gamberini, 2011; Eghbali et al., 2023b). The advent of iron-chelating medications has significantly enhanced the life expectancy of patients in recent decades as these drugs aid in the removal of excess iron from the body and mitigate iron accumulation in tissues (Crichton et al., 2019). Currently, clinical practice involves the use of three iron-chelating drugs: deferoxamine (DFO), which requires administration via subcutaneous, intravenous, or intramuscular routes due to limited oral absorption (Wali et al., 2004); deferiprone (DFP); and deferasirox (DFX). DFX is accessible in various formulations, including Exjade^®^ dispersible tablets (DFX-DT) for oral intake once daily, fully dissolved in liquids and ingested 30 min before meals on an empty stomach, and Jadenu^®^ granules (DFX-GF) (Cheng et al., 2018; Falahati et al., 2022) and Jadenu^®^ film-coated tablets (DFX-FCT) in the Iranian pharmaceutical market. Jadenu^®^, a generic alternative to Jadenu^®^ from Novartis, and unlike Exjade^®^, does not necessitate dissolution in liquids and can be consumed in a single step, with or without a light meal (Yassin et al., 2018a). The efficacy of Exjade^®^ has been well-documented in TM patients (Kattamis et al., 2018). Moreover, the enhanced palatability, compliance, elevated bioavailability, and reduced gastrointestinal (GI) side effects of Jadenu^®^ offer promising outcomes for long-term treatment (Cheng et al., 2018; Taher et al., 2018). DFX-FCT, prescribed according to body weight, is available in three dosage strengths (90 mg, 180 mg, and 360 mg) (Taher et al., 2018).

Exjade^®^ cannot be swallowed and should be taken on an empty stomach and dissolved in water or juice. This method of administration results in a smaller amount of the dose being absorbed by the body. In addition, about a third of patients report the bad taste of this medicine (Waldmeier et al., 2010). The most common side effect of this drug is digestive problems, with approximately 10%–33% of patients using this drug reporting digestive problems, including diarrhea, nausea, vomiting, and heartache. It has been shown that although most of the patients can tolerate this drug, approximately 7% discontinue its due to digestive problems (Gattermann and Rachmilewitz, 2011; Angelucci et al., 2014). Therefore, a new formulation of DFX oral drug has been launched under the brand name Jadenu^®^. This drug is the only oral chelator available in the market that can be swallowed whole, without the need to for dissolution in liquids. In this study, we compared the effect of DFX granules (Exjade^®^) and the oral form (Jadenu^®^) on patient satisfaction and convenience, cardiac/liver MRI T2 levels*, serum ferritin levels, and biochemical profiles in patients with thalassemia major.

## Materials and methods

### Patients

In a randomized clinical trial conducted between May 2021 and April 2023, a total of 114 individuals diagnosed with β-thalassemia major were carefully selected at Amir-Kabir Hospital in Arak, Iran. The diagnosis of thalassemia was conducted using accepted diagnostic methods. All patients with thalassemia major received iron chelation therapy concomitantly with packed red blood cells every 3–4 weeks.

Patients were categorized into two groups using a simple randomization method (random number generation software, with group 1 assigned to patients with odd numbers and group 2 assigned to those with even numbers). Before the study commenced, the department head (who was not affiliated with the principal investigators) conducted the randomization process.

All patients had received DFX before entering the study. They were divided into two groups: one receiving 20–30 mg/kg/day of Exjade^®^ (Novartis) for 6 months and the other receiving the same dose of Jadenu^®^ (Nano Hayat Daru Pharmaceutical Company, Tehran, Iran; GTIN: 06262849300336; IRC: 3.7616388636e+015) orally for 6 months. The entire study adhered strictly to the ethical guidelines outlined in the approved ethical protocol by the Research Ethics Committee of Arak University of Medical Sciences (IR.ARAKMU.REC.1399.250). Following the acquisition of informed consent, individuals aged between 2 and 50 years with thalassemia major were included in the study. A structured questionnaire was employed to collect pertinent patient information. All patients were already receiving Exjade^®^ and Jadenu^®^ prior to the commencement of the study, which lasted for 9 months. Throughout this period, the patients were meticulously monitored and evaluated. Notably, the study was conducted in full compliance with the principles outlined in the Declaration of Helsinki (Riis, 2003). Demographic information, side effects, taste satisfaction, and consumption method were evaluated through a written questionnaire.

### Inclusion and exclusion criteria

Inclusion criteria encompassed the following parameters: patients diagnosed with thalassemia major who had been treated with Exjade^®^ and Jadenu^®^, a serum ferritin above 1,000 μg/mL, initiation of transfusion therapy after the age of 2, and commencement of iron chelation therapy prior to reaching 5 years of age. The study’s exclusion criteria involved patients with a GFR ˂40 mL/min, known allergies to DFX group drugs, simultaneous use of other iron chelators, and conditions such as hypertension, renal failure, pyrexia, severe infections, uncontrolled hyperglycemia, dissatisfaction with treatment, hepatic disorders, inflammatory conditions, protein in the urine, intolerance to oral medication, hepatitis B, hepatitis C, patients unwilling to continue participation in the research, and those who declined to complete the study. A total of nine thalassemia major patients were disqualified from the research due to failure to meeting the specified criteria.

### Evaluation of liver and heart iron overload

The degree of cardiac and hepatic hemosiderosis of patients was assessed using the routine T2 * MRI method (Siemens Healthineers, Germany). The serum ferritin level was assessed using an ELISA Kit (Pishtaz Teb Ltd., Tehran, Iran). In addition, to check liver function during 6 months, serum alanine aminotransferase (ALT), aspartate aminotransferase (AST), and alkaline phosphatase (ALK) levels of both groups were measured in a single laboratory under the same conditions every 2 months. The level of liver enzymes was measured by the photometric method (Pars Azmun Company, Iran) using an auto-analyzer (Auto-analyzer BT3000, Biotechnica, Italy). All measurements were performed in an accredited medical laboratory.

Furthermore, the satisfaction of the patients with the iron chelator was assessed using a 5-point Likert scale, which comprised the following options: very satisfied, satisfied, neutral, dissatisfied, and very dissatisfied. Patients were also asked to rate their level of satisfaction with the method of taking the medicine on a separate 5-point Likert scale with the following options: very comfortable, comfortable, neutral, unpleasant, and very uncomfortable.

### Statistical analysis

Data were analyzed using SPSS version 22 software (SPSS Inc., Chicago, IL). Numerical variables are presented as the mean and standard deviation (SD). The two groups were compared using Pearson’s χ^2^ test for qualitative variables and the independent t-test for quantitative variables. Quantitative variables at different months in each group were compared using the paired t-test. Mean values of ALT, AST, and ALK before and after intervention were compared within groups using the paired *t*-test and repeated measure ANOVA. *p* < 0.05 was considered statistically significant.

## Results

**FIGURE 1:**
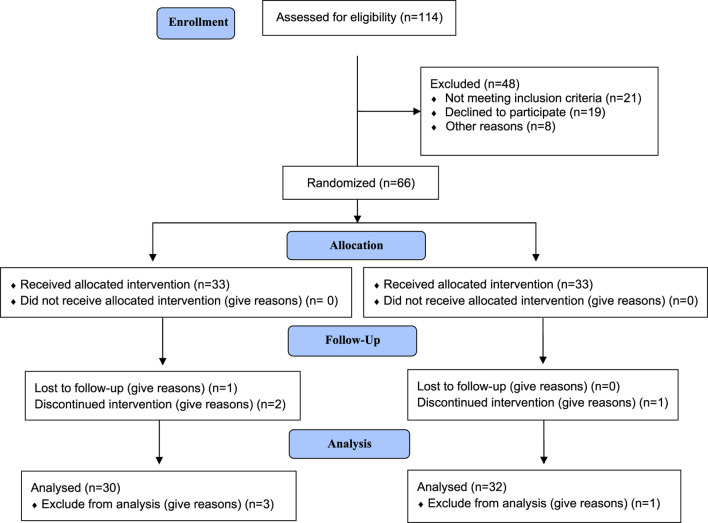
Flowchart of the study.

A total of 62 patients (32 patients in the Exjade^®^ group and 30 patients in the Jadenu^®^ group) completed the study, as presented in the CONSORT diagram (Figure 1). Of the 62 patients with β-thalassemia major, 29 (46.7%) were male and 33 (53.2%) were female.

Over the previous 5 years, the average hemoglobin concentration was 7.6 ± 3.8 g/dL. Every patient received a transfusion of 10–15 mL of packed red blood cells per kilogram of body weight, depending on their Hb concentration. The mean ± SD duration of receiving iron-chelating agents was 12.3 ± 4.4 years, with a range from 6 to 25 years. All patients had normal fasting blood sugar during the frequent routine testing over the past 5 years. Weights ranged from 29 to 65 kg, with a mean of 48.3 kg. Seventy-one percent of patients received blood transfusions every 2 weeks, and the rest of patients received blood transfusions every 3 or 4 weeks.

The mean ± SD age of the patients at diagnosis was 26.7 ± 6.5 years (range 2–50 years), and the mean ± SD follow-up time of patients was 5.3 ± 2.5 months. The mean Jadenu^®^ dose administered was 26.5 ± 5.1 mg/kg/day, and the mean Exjade^®^ dose administered was 25.9 ± 5.8 mg/kg/day. Demographic data of the two groups are shown in Table 1.

**TABLE 1:** Comparison of demographic characteristics of the patients with beta thalassemia.

Variable	Exjade^®^ group (n = 32)	Jadenu^®^ group (n = 30)	*p*-value
Age (year)	26.4 ± 7.8^a^	27.1 ± 10.1^a^	0.421^b^
Gender, n (%)			
Male	17 (53.1)	12 (40.0)	0.502^c^
Female	15 (46.9)	18 (60.0)	
BMI, kg/m^2^	20.1 ± 4.3	18.8 ± 3.7	0.287
Height (cm)	126.7 ± 6.7	124.4 ± 9.1	0.624
Weight (kg)	24.7 ± 4.3	25.6 ± 3.8	0.863
Splenectomy, n (%)	18 (56.2)	16 (53.3)	0.798

^a^ Mean and standard deviation.

^*b*^ *p*-values were determined using the Student’s t-test.

^c^ *p*-values were determined using the Pearson’s χ2 test. n, number of patients and BMI, body mass index.

In the present study, after 6 months of taking the studied drugs, satisfaction with the taste and convenience of these two types of drugs were evaluated (Table 2). The results showed that 53.3% of the patients in the Jadenu^®^ group were satisfied with the taste of the medicine (very satisfied/satisfied). On the other hand, in the Exjade^®^ group, only 12.5% of patients were satisfied with the taste, and the difference between the two groups was statistically significant (*p* = 0.001).

**TABLE 2:** Comparison of satisfaction and convenience with Jadenu^®^ and Exjade^®^ in the patients with beta thalassemia.

Variable	Exjade^®^ group (n = 32)	Jadenu^®^ group (n = 30)	*p*-value
Rating of satisfaction with taste, n (%)			
Very satisfied/satisfied	4 (12.5)	16 (53.3)	0.001
Neutral	8 (25.0)	10 (33.3)	
Dissatisfied/very dissatisfied	20 (62.5)	4 (13.4)	
Rating of convenience, n (%)			
Very satisfied/satisfied	9 (28.1)	12 (40.0)	0.047
Neutral	9 (28.1)	13 (43.3)	
Dissatisfied/very dissatisfied	14 (43.8)	5 (16.7)	
Gastrointestinal complications, n (%)	9 (28.1)	2 (6.6)	0.028

The results showed that 40% of the patients in the Jadenu^®^ group were satisfied with the method of taking the medicine (very satisfied/satisfied). However, in the Exjade^®^ group, only 28.1% of patients were satisfied with the taste, and the difference between the two groups was statistically significant (*p* = 0.047).

GI complications were also evaluated in patients of both groups based on diarrhea, anorexia, constipation, and vomiting. GI complications in the Exjade^®^ group were significantly higher than those in the Jadenu^®^ group (*p* = 0.028).

The cardiac MRI T2* level at baseline (at the beginning of the study) in the Exjade^®^ and Jadenu^®^ groups was 22.1 and 19.9 ms, respectively (Table 3). A comparison of cardiac T2* levels at baseline between the two studied groups showed no significant difference (*p* = 0.357). Cardiac MRI T2*, 6 months after the start of treatment, in the Exjade^®^ and Jadenu^®^ groups was 23.1 and 22.70 ms, respectively. A comparison of the cardiac MRI T2* level between the two studied groups showed no significant difference (*p* = 0.851).

**TABLE 3:** Cardiac and liver MRI T2* alteration in patients with beta thalassemia major receiving Exjade^®^ or Jadenu^®^.

Variable	Exjade^®^ group Mean ± SD	Jadenu^®^ group Mean ± SD	*p*-value
Cardiac MRI T2* (millisecond)			
baseline	22.1 ± 9.9	19.9 ± 8.8	0.357
after 6 months	23.1 ± 8.4	22.7 ± 6.5	0.851
Liver MRI T2* (millisecond)			
baseline	7.5 ± 7.1	7.7 ± 8.2	0.919
after 6 months	11.2 ± 8.7	11.7 ± 8.2	0.759

MRI T2*, magnetic resonance imaging T2*.

In addition, hepatic MRI T2* at baseline (at the beginning of the study) in the Exjade^®^ and Jadenu^®^ groups was 7.5 and 7.7 ms, respectively. A comparison of hepatic MRI T2* before treatment between the two study groups using the independent t-test showed no significant difference (*p* = 0.919). Hepatic MRI T2*, 6 months after the start of treatment, in the Exjade^®^ and Jadenu^®^ groups was 11.2 and 11.7 ms, respectively. A comparison of hepatic MRI T2* between the two studied groups showed no significant difference (*p* = 0.759).

**FIGURE 2:**
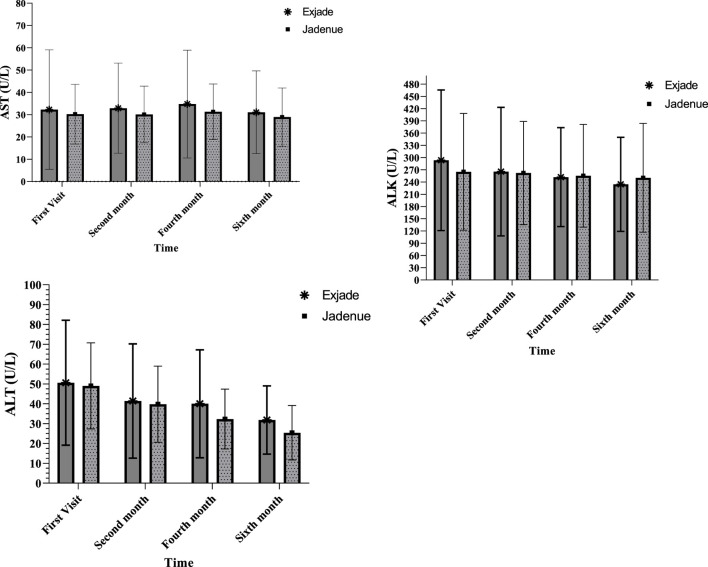
Comparison of AST, ALT, and ALK levels between two study groups treated with Exjade^®^ and Jadenu^®^ at four different times.

Biochemical parameters of patients with β‐thalassemia major are presented in Figure 2. The highest level of AST was observed in 4 months from the start of treatment, and this value was 34.7 and 31.2 in the Exjade^®^ and Jadenu^®^ groups, respectively, which was in the normal range. A comparison of the AST level using a repeated measure ANOVA test showed no significant difference between treatment groups at different times. In addition, there was no significant difference between different times in each group, and the time-group effect was also not significant. A comparison of ALT and ALK levels showed that ALT levels decreased significantly compared to baseline in both groups 6 months after the start of treatment. It was also observed that there is no significant difference between treatment groups at different times. In addition, there was a significant difference between different times in each group, and the ALT level decreased over time.

Serum ferritin levels ranged from 502–5567 ng/mL, with a mean of 1,633.1 ng/mL. Over the previous 5 years, the average hemoglobin concentration was 7.6 ± 3.8 g/dL. Every patient received a transfusion of 10–15 mL of packed red blood cells per kilogram of body weight, depending on their Hb concentration.

The mean ± SD ferritin level of the patients in the Jadenu^®^ group at baseline was 1,421.1 ± 836.5 ng/mL. Its levels reduced from 1,338.7 ± 498.9 ng/mL after 3 months to 1,206.3 ± 532.7 ng/mL at the end of the study; however, the decrease in serum ferritin levels was not significant (*p* > 0.05).

In the Exjade^®^ group, the mean ± SD ferritin level of patients at baseline was 1,376.1 ± 987.6 ng/mL. Its levels reduced from 1,217.4 ± 489.4 ng/mL after 3 months to 1,100.2 ± 670.1 ng/mL at the end of the study; however, the decrease in serum ferritin levels was not significant (*p* > 0.05). So, mean ferritin levels decreased steadily in both groups during the study period. There was no significant difference between the Jadenu and Exjade groups in reducing ferritin levels (*p* > 0.05).

## Discussion

This study aimed to compare the effect of deferasirox granular (Exjade^®^) with oral form (Jadenu^®^) on iron deposition in the heart and liver in patients with thalassemia major. The results of this study showed that GI complications were significantly higher in the Exjade^®^ group than in the Jadenu^®^ group. In addition, patient satisfaction and convenience were significantly higher in the Jadenu group. In addition, although the effect of Jadenu on the reduction of heart and liver iron overload and liver enzymes was greater, this difference between the two studied groups was not significant.

Compared to Exjade^®^, Jadenu^®^ offers greater convenience and flexibility of use. Exjade^®^ is a once-daily oral iron chelator that was developed to address the need for a long-acting chelator. One-third of patients consider Exjade^®^, in its oral suspension form, unpleasant. In addition, approximately one-quarter of patients experience mild-to-moderate GI symptoms, which may present additional challenges, particularly in the younger and older age ranges. The new Jadenu^®^ tablet formulation was developed to overcome intolerance issues and is the only once-daily oral iron chelator that can be swallowed with a light meal, without the need for pre-dose suspension (Andrews, 1999).

The most common side effect of DFX tablets for oral suspension is GI upset, with 10%–33% of patients experiencing abdominal pain, diarrhea, nausea, and/or vomiting (Neufeld, 2006). However, most patients were able to tolerate these side effects although 7% of patients cited GI side effects as a reason for treatment discontinuation (Fisher et al., 2013). Because DFX can cause nephrotoxicity and proteinuria, creatinine should be measured twice before starting treatment and then monitored monthly after the start of treatment (Neufeld, 2006; Yassin et al., 2018b). The new formulation of DFX (Jadenu^®^) has been able to improve patient satisfaction and, thus, adherence to treatment due to its ease of use (Goldberg et al., 2013).

Yassin et al. (2018b) reported that 66.6% of patients with thalassemia major found the palatability of Jadenu^®^ improved compared to Exjade^®^. In addition, approximately 25% of patients in their study reported minor GI complaints during Jadenu^®^ treatment, but none of these symptoms led to treatment discontinuation. During the study period, they did not record significant changes in serum creatinine concentration, albumin level, urinalysis, glucose levels, or thyroid function (Yassin et al., 2018b). The results of this study are in line with those of our study. In our study, 23 out of 30 patients treated with Jadenu^®^ reported improvement in taste. In addition, 2 out of 30 patients had GI complaints.

Various studies have investigated the effect of Exjade^®^ on the amount of iron overload on different organs, including the heart and liver, but no study has compared the effect of two drugs, Exjade^®^ and Jadenu^®^, on MRI T2 changes in the heart and liver of thalassemia patients. For example, Pathare et al. (2010) showed that the granular form of DFX (Exjade^®^) played a significant role in reducing or preventing the accumulation of iron in the heart and liver. They also reported that iron in the heart is removed at a slower rate than that in the liver (Pathare et al., 2010). In another study, the results showed that the granular form of DFX drug is effective in reducing iron overload and improving heart MRIT2, and the use of this drug has been associated with reducing heart failure in the long term in these patients (Chalmers and Shammo, 2016). Similar to the results of these studies, in our study, the consumption of DFX was associated with a decrease in liver MRI T2 and cardiac MRI T2 levels in the two study groups, with the difference that in our study, two forms of the drug were compared, while in the mentioned studies, only the effect of granular form was investigated.

Tinsley and Hoehner-Cooper (2018) stated that Jadenu^®^ offers greater convenience and flexibility compared to Exjade^®^. In addition, because Jadenu^®^ is more bioavailable compared to Exjade^®^, the recommended dose of DFX-FCT is approximately 30% lower than that of DFX-DT. Compared to Exjade^®^, Jadenu^®^ has fewer food restrictions: while Exjade^®^ must be taken on an empty stomach at least 30 min before a meal, Jadenu^®^ can be taken on an empty stomach or with a light meal. Exjade^®^ needs to be dissolved in a limited number of liquids (water, orange juice, or apple juice) and the amount of liquid must be measured, but Jadenu^®^, on the other hand, can be swallowed whole with water or other beverages and its consumption is easy (Tinsley and Hoehner-Cooper, 2018).

In another study that compared two formulations of DFX in patients with transfusion-dependent beta-thalassemia, the results showed that patients reported greater satisfaction with the palatability of Jadenu^®^ and less concern about GI symptoms with Jadenu^®^ than with the original formulation. In addition, patients showed better compliance and continued treatment for a longer period of time and experienced a greater decrease in serum ferritin (Taher et al., 2017).

One of the limitations of this study is the relatively small sample size that only 62 patients participated. This small sample size may limit the generalizability of the findings to a larger population. Therefore, it is suggested to conduct a study with a larger sample size in the future.

In addition, another limitation of this study is the evaluation of the effects of Jadenu^®^ and Exjade^®^ over a period of 6 months, which may not be enough to fully evaluate the effectiveness and long-term safety of the drugs. Therefore, to evaluate the mentioned issue, it is suggested to first conduct an animal study, followed by human trials. Moreover, the duration of drug evaluation should be increased to more than 6 months.

In conclusion, the results showed that patients who received Jadenu^®^ had better treatment compliance, which is a promising result for continued treatment, although further long-term evaluation of Jadenu^®^ is needed to support these results. By simplifying the administration of the new DFX formulation (Jadenu^®^), it is aimed to enhance patient satisfaction, thereby improving adherence to treatment. Jadenu^®^ offers several advantages to patients, including the ability to be taken on an empty stomach, a better taste, and fewer gastrointestinal tolerability concerns. Overall, patient satisfaction is higher with Jadenu^®^, which may not only improve adherence but also reduce the frequency and severity of complications associated with iron overload. This, in turn, may help mitigate cardiovascular and hepatic complications from iron overload in the long term.

## Data Availability

The raw data supporting the conclusions of this article will be made available by the authors, without undue reservation.
